# Tumor Growth Remains Refractory to *Myc* Ablation in Host Macrophages

**DOI:** 10.3390/cells11244104

**Published:** 2022-12-17

**Authors:** Riley J. Morrow, Amr H. Allam, Josh Konecnik, David Baloyan, Christine Dijkstra, Moritz F. Eissmann, Saumya P. Jacob, Megan O’Brien, Ashleigh R. Poh, Matthias Ernst

**Affiliations:** The Olivia Newton-John Cancer Research Institute and School of Cancer Medicine, La Trobe University, Heidelberg, VIC 3084, Australia

**Keywords:** Myc, gastrointestinal cancer, myeloid cells, macrophages

## Abstract

Aberrant expression of the oncoprotein c-Myc (Myc) is frequently observed in solid tumors and is associated with reduced overall survival. In addition to well-recognized cancer cell-intrinsic roles of Myc, studies have also suggested tumor-promoting roles for Myc in cells of the tumor microenvironment, including macrophages and other myeloid cells. Here, we benchmark *Myc* inactivation in tumor cells against the contribution of its expression in myeloid cells of murine hosts that harbor endogenous or allograft tumors. Surprisingly, we observe that *LysM*^Cre^-mediated *Myc* ablation in host macrophages does not attenuate tumor growth regardless of immunogenicity, the cellular origin of the tumor, the site it develops, or the stage along the tumor progression cascade. Likewise, we find no evidence for *Myc* ablation to revert or antagonize the polarization of alternatively activated immunosuppressive macrophages. Thus, we surmise that systemic targeting of Myc activity may confer therapeutic benefits primarily through limiting Myc activity in tumor cells rather than reinvigorating the anti-tumor activity of macrophages.

## 1. Introduction

Macrophages are a major component of the tumor microenvironment and are associated with a poor prognosis in most solid malignancies. Depending on their activation status, macrophages can exert dual influences on tumorigenesis by either enhancing immune cell activation or by antagonizing cytotoxic immune responses. Classically activated M1 macrophages are important drivers of anti-tumor immunity by mediating antibody-dependent cellular cytotoxicity and phagocytosis, amplifying innate and adaptive immune surveillance, and promoting tumor necrosis [1]. In contrast, alternatively activated M2 macrophages play a pivotal role in tumor initiation and progression by contributing to angiogenesis, immune suppression, and extracellular matrix remodeling, as well as resistance to chemotherapy and immune checkpoint inhibitors [2]. For this reason, therapies aimed at reprogramming tumor-associated macrophages (TAMs) away from an alternatively activated endotype represent a promising strategy for anti-cancer therapy.

The oncoprotein c-Myc (Myc) is the prototypical member of the Myc family of transcription factors that regulate a broad spectrum of biological processes, including proliferation, angiogenesis, tissue remodeling, metabolism, and hematopoiesis [3,4,5]. Although Myc activity is tightly regulated in normal cells, deregulation of Myc is observed in up to 70% of tumors [6] and is associated with a poor prognosis and reduced patient survival [7]. In support of its tumor cell-intrinsic role, the inactivation of Myc in cancer cells results in tumor regression by promoting proliferative arrest, cellular senescence, and the induction of apoptosis [8]. Meanwhile, Myc also plays a major tumor cell-extrinsic role by facilitating immune evasion through decreased expression of MHC I and upregulation of inhibitory cytokines and immune checkpoint proteins [9,10,11]. Notably, Myc has been suggested to transcriptionally regulate the expression of genes associated with alternative macrophage polarization [12,13,14], while the inhibition of Myc skews macrophages towards a classically activated inflammatory endotype [15]. These findings suggest that inhibition of Myc in TAMs may offer a therapeutic opportunity to curb tumor growth by reprogramming the immunosuppressive tumor microenvironment conferred by alternatively activated macrophages.

In this study, we explored as a first proof-of-principle assessment whether conditional ablation of *Myc* in host macrophages impairs tumor growth by reducing the polarization of alternatively activated TAMs in vivo. Unexpectedly, we found no evidence that conditional ablation of *Myc* expression in macrophages reduces tumor growth across four complementary pre-clinical models. Our observations suggest that systemic targeting of Myc activity may therefore confer therapeutic benefits primarily through limiting Myc activity in tumor cells rather than suppressing Myc in TAMs.

## 2. Materials and Methods

### 2.1. Mice

Age- and sex-matched mice were bred and maintained in specific pathogen-free facilities at La Trobe University and the Austin Hospital, Australia. The *LysM*^Cre/+^ strain [16] was crossed with the *Myc*^fl/fl^ strain [17] to generate *LysM*^Cre/+^;*Myc*^fl/fl^ and *LysM*^+/+^;*Myc*^fl/fl^ mice [14]. Where indicated, *LysM*^Cre/+^;*Myc*^fl/fl^ and *LysM*^+/+^;*Myc*^fl/fl^ mice were additionally crossed with *Gp130*^F/F^ mice [18] to generate *LysM*^Cre/+^;*Myc*^fl/fl^;*Gp130*^F/F^ and *LysM*^+/+^;*Myc*^fl/fl^;*Gp130*^F/F^ animals. The *Tff1*^CreERT2^ strain [19] was crossed with *Myc*^fl/fl^;*Gp130*^F/F^ animals to generate *Tff1*^CreERT2^;*Myc*^fl/fl^;*Gp130*^F/F^ compound mutant mice. All animal studies were approved and conducted in accordance with the Animal Ethics Committee at La Trobe University and the Olivia Newton-John Cancer Research Institute/Austin Hospital.

### 2.2. Tumor Models

The mouse MC38 colon cancer and B16F10 melanoma cell lines were maintained in DMEM/F12 (Gibco #11320033, Waltham, MA, USA) supplemented with 10% FCS at 37 °C with 10% CO_2_. Cell lines tested negative for mycoplasma. Six-week-old *LysM*^Cre/+^;*Myc*^fl/fl^ and *LysM*^+/+^;*Myc*^fl/fl^ mice were subcutaneously injected with 1 × 10^6^ MC38 or 2 × 10^5^ B16F10 cells into the right flank. Mice were collected at 2 weeks following tumor cell injection.

Mouse gastric tumor organoids were derived from gastric adenocarcinomas of *Kras*^G12D/+^;*Pik3ca*^H1047R/+^;*Trp53*^R172H/+^ (KPT) mutant mice (M.F. Eissmann; unpublished) and maintained as previously described [20,21]. Six-week-old *LysM*^+/+^;*Myc*^fl/fl^ and *LysM*^Cre/+^;*Myc*^fl/fl^ littermates were subcutaneously injected with 900 organoids into the right flank. Mice were collected at 3 weeks following tumor organoid injection.

*LysM*^+/+^;*Myc*^fl/fl^;*Gp130*^F/F^ and *LysM*^Cre/+^;*Myc*^fl/fl^;*Gp130*^F/F^ animals were euthanized at 100 days of age. Stomachs were dissected longitudinally along the greater curvature, and gastric tumors were dissected and weighed.

### 2.3. Ablation of Gastric Epithelial Cells via Tamoxifen Treatment

Ninety-day-old *Tff1*^CreERT2^;*Myc*^fl/fl^;*Gp130*^F/F^ mice were administered a total of 6 mg tamoxifen (dissolved in 10% ethanol and 90% sunflower oil) via intraperitoneal injection (1mg/mL per dose; 2 doses a day over 3 consecutive days). Mice were euthanized 7 days following the last tamoxifen injection. Stomachs were dissected longitudinally along the greater curvature, and gastric tumors were dissected and weighed.

### 2.4. Immunofluorescence

Paraffin-embedded formalin-fixed sections were dewaxed in xylene and rehydrated in ethanol. Antigen retrieval was performed by incubating slides in EDTA buffer (pH 9) for 20 min at 95 °C. Sections were immersed in 3% H_2_O_2_ for 10 min at room temperature to inhibit endogenous peroxidase activity, washed in TBST, then blocked in 2% bovine serum for 1 h at room temperature. Monoplex staining with primary antibodies was performed using the OPAL 7 color kit (Akoya Biosciences, Marlborough, MA, USA) as previously described [22]. Following incubation with HRP-conjugated secondary antibodies, slides were incubated with individual tyramide signal amplification (TSA)-conjugated fluorophores (Akoya Biosciences, Marlborough, MA, USA) for 10 min at room temperature then washed with TBST. Slides were mounted with Fluoromount-G (Thermofisher #00-4958-02, Waltham, MA, USA) and scanned using a 20× objective on the Vectra^®^ 3 automated quantitative pathology imaging system (Akoya Biosciences, Marlborough, MA, USA). inForm software (Version 2.2, Akoya Biosciences, Marlborough, MA, USA) was used to build a spectral library using monoplex scans.

Following the identification of optimal staining parameters for monoplex staining, staining of the full multiplex panel was performed. First, staining for Myc (Abcam #ab32072, Waltham, MA, USA) was performed using the steps outlined above. After incubation with TSA dye, slides were washed in TBST, and antigen retrieval was performed again to remove the primary and secondary antibody complex. Next, F4/80 staining (Cell Signaling #D2S9R, Danvers, MA, USA) was performed. After staining with TSA dye, slides were washed in TBST. Sections were then incubated with spectral DAPI (Akoya Biosciences, Marlborough, MA, USA), washed in dH_2_O, and mounted with Fluoromount-G. Slides were scanned using a 20× objective on the Vectra^®^ 3 automated quantitative pathology imaging system. inForm software (Version 2.2, Akoya Biosciences, Marlborough, MA, USA) was used to analyze images.

### 2.5. Flow Cytometry

Tumors were cut into 1 mm pieces and digested in Collagenase/Dispase (Roche #11097113001, Basel, Switzerland) and DNase I (Roche #10104159001, Basel, Switzerland) diluted in Ca^2+^/Mg^2+^-free HBSS media (Gibco #14170112, Waltham, MA, USA) plus 10% FCS for 30 min at 37 °C [23]. Samples were vortexed for 15 s, then filtered and washed in PBS plus 10% FCS. After incubation with Fc block (ThermoFisher #14-9161-73, Waltham, MA, USA) on ice for 10 min, cells were stained with fluorophore-conjugated primary antibodies for 20 min on ice in the dark, washed twice, and re-suspended in PBS supplemented with 10% FCS.

The antibodies used included CD45.2 (Clone 30-F11; BioLegend #103116, San Diego, CA, USA), F4/80 (Clone BM8; BioLegend #123114, San Diego, CA, USA), CD11b (Clone M1/70; BioLegend #101208, San Diego, CA, USA), Ly6G (Clone 1A8, BD Biosciences #560602, Franklin Lakes, NJ, USA), Ly6C (Clone HK.4, eBioscience #48-5932-82, Waltham, MA, USA), TCRβ (Clone H57-597; BD Biosciences #553170, Franklin Lakes, NJ, USA) and CD8a (Clone 53-6.7; BioLegend #100712, San Diego, CA, USA).

Flow cytometry was performed on a BD FACS Aria III cell sorter and analyzed using FlowJo software (Version 10, Oregon, USA). Background fluorescence was estimated using isotype controls, fluorescent-minus-one controls, and unstained controls. Dead cells were excluded by Sytox Blue (ThermoFisher #S34857, Waltham, MA, USA) staining.

### 2.6. RNA Extraction and qPCR

RNA extraction was performed using the RN-easy Micro Plus kit (Qiagen #74034, Hilden, Germany) and RN-easy Mini Plus kit (Qiagen #74134, Hilden, Germany) for FACS-isolated cells and whole tumors, respectively. cDNA from FACS-isolated cells was generated using the SuperScript™ IV First-Strand Synthesis System (ThermoFisher #18091050, Waltham, MA, USA), and cDNA from tumors was generated using the High-Capacity cDNA Reverse Transcription Kit (ThermoFisher #4368814, Waltham, MA, USA).

qPCR analysis on each biological sample was performed using technical replicates with Taqman^®^ Real-Time PCR Master mix and probes (ThermoFisher #4352042, Waltham, MA, USA). Samples were run on the Viia7 Real-Time PCR System for 40 cycles (95 °C for 15 s, 60 °C for 1 min) with an initial holding stage (95 °C for 3 min). Fold changes in gene expression were obtained using the 2−ΔΔCT method [24].

The Taqman probes used were mouse *18s* (Mm04277571_s1), *Gapdh* (Mm99999915_g1), *Hprt* (Mm03024075_m1) *Myc* (Mm00487804_m1) *Il4* (Mm00445259_m1), *Il10* (Mm01288386_m1), *Il13* (Mm00434204_m1), *Tgfβ* (Mm01227699_m1), *Vegfα* (Mm00437306_m1), *Arg1* (Mm00475988_m1), *Ym1* (Mm00657889_mH), *Il1β* (Mm00434228_m1), *Tnf* (Mm00443258_m1), *Nos2* (Mm00440502_m1), *GzmB* (Mm00442837_m1), and *Prf1* (Mm00812512_m1).

### 2.7. Western Blot Analysis

Tumor protein lysates were resolved on 4–12% NuPAGE Bis-Tris gels [25,26]. Following dry transfer using an iBlot 2 (ThermoFisher, Waltham, MA, USA), PVDF membranes were blocked in Intercept Blocking Buffer (LI-COR Biosciences #927-70001, Lincoln, NE, USA) for 1 h at room temperature. Membranes were incubated overnight in anti-Myc antibody (Abcam #ab32072, Waltham, MA, USA) and anti-actin antibody (loading control; Sigma #A228, Saint Louis, MO, USA) at 4 °C. The next day, blots were washed twice in TBST, then stained with fluorescent-conjugated secondary antibodies (LI-COR Biosciences #926-32221 and #926-32210, Lincoln, NE, USA) for 1 h at room temperature. After two additional washes in TBST, signals were detected using the Odyssey Infrared Imaging System (LI-COR Biosciences, Lincoln, NE, USA).

### 2.8. Isolation of Bone-Marrow-Derived Macrophages

Bone marrow was harvested from the femur and tibia of mice by flushing with sterile PBS as previously described [25,26]. Cells were washed twice in PBS and filtered through a 70 μm sieve. The single-cell suspension was then cultured in Macrophage Media (DMEM/F12 supplemented with 10% FCS and 20% L929 conditioned media). To fully differentiate bone-marrow-derived macrophages, cells were cultured for 7 days with fresh media changed every 3 days. Adherent macrophages were detached from plates using a cell scraper and processed for downstream analysis.

### 2.9. Isolation of Peritoneal Macrophages

The peritoneal cavity of mice was flushed with 5 mls of PBS supplemented with 3% FCS and gently massaged to detach immune cells. The cell suspension was aspirated and pelleted by centrifugation for downstream FACS sorting to isolate CD45^+^CD11b^+^F4/80^+^Ly6C^−^LygG^−^ macrophages.

### 2.10. Isolation of Splenic Macrophages

Spleens were mashed through a 70 µm filter, resuspended in 30 mls of PBS, and pelleted by centrifugation. Cells were incubated in red cell lysis buffer for 5 min, washed in PBS plus 10% FCS, and pelleted by centrifugation for downstream FACS sorting to isolate CD45^+^CD11b^+^F4/80^+^Ly6C^−^LygG^−^ macrophages.

### 2.11. Quantification and Statistical Analysis

All experiments were performed at least twice with a minimum of three age- and sex-matched mice per group. The specific n (number of animals) used per cohort is indicated in the respective figure legends and shown as individual data points. No data were excluded from the analysis. Tumor weights were recorded by an independent assessor who was blinded to the experimental conditions. Statistical analysis was conducted using GraphPad Prism Software (Version 8). Comparisons between two mean values were performed with a 2-tailed Student’s *t*-test. A *p* value of less than 0.05 was considered statistically significant.

## 3. Results

### 3.1. Myc Expression Is Reduced in Macrophages of LysM^Cre/+^;Myc^fl/fl^ Mice

**Figure 1:**
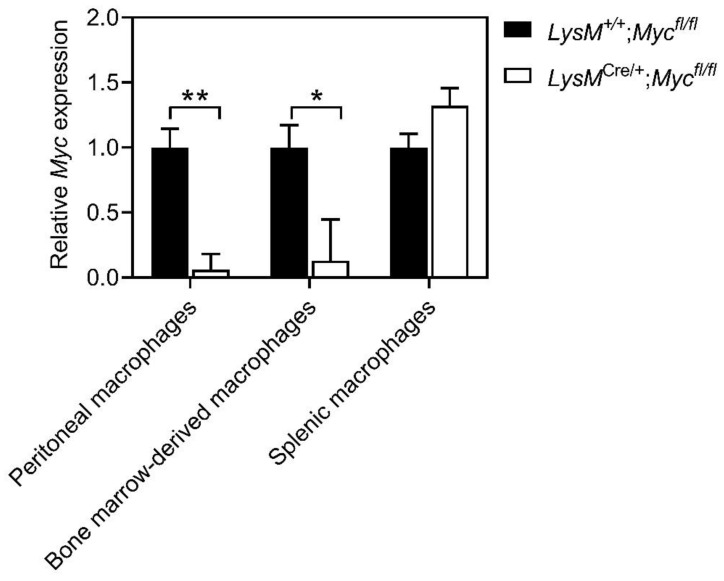
***LysM*^Cre^-mediated reduction in *Myc* expression in mature macrophages.** Expression of *Myc* in peritoneal, bone-marrow-derived, and splenic macrophages isolated from *LysM*^+/+^;*Myc*^fl/fl^ and *LysM*^Cre/+^;*Myc*^fl/fl^ mice. n ≥ 3 mice per group. Data represent mean ± SEM; * *p* < 0.05, ** *p* < 0.01, with statistical significance determined by an unpaired Student’s *t*-test.

The transgenic *LysM*^Cre/+^ knock-in strain enables conditional depletion of *Myc* gene expression in macrophages of *Myc*^fl/fl^ mice due to lox(p) sites flanking exons 2 and 3 of *Myc* [27]. Consequently, the abundance of functional Myc protein in mature macrophages of *LysM*^Cre/+^;*Myc*^fl/fl^ mice is reduced by up to 90% [14]. To validate these observations, we performed qPCR analysis on peritoneal and bone-marrow-derived macrophages isolated from *LysM*^Cre/+^;*Myc*^fl/fl^ mice. We observed an 80–90% reduction in *Myc* expression in these cells compared to cells purified from *LysM*^Cre^ transgene-deficient *LysM*^+/+^;*Myc*^fl/fl^ littermate controls (Figure 1). Consistent with previous observations of limited Cre recombinase activity conferred by *LysM*^Cre^ in splenic myeloid cells [27], we confirmed a lack of quantitative *Myc* ablation in these cells (Figure 1).

### 3.2. Tumor Growth Remains Refractory to Myc Ablation in Host Macrophages

**Figure 2:**
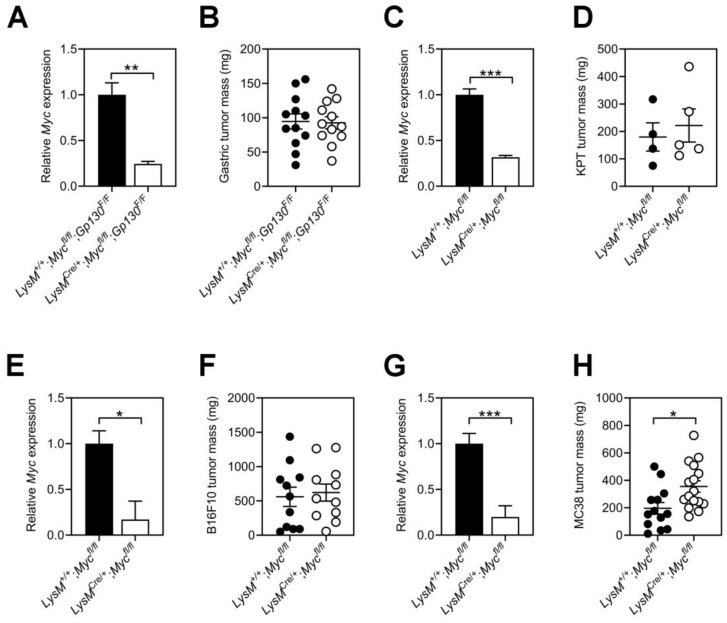
**Tumor growth remains refractory to *Myc* ablation in host macrophages.** (**A**) *Myc* gene expression in gastric tumors of *LysM*^+/+^;*Myc*^fl/fl^;*Gp130*^F/F^ and *LysM*^Cre/+^;*Myc*^fl/fl^;*Gp130*^F/F^ mice. n = 4 mice per group. (**B**) Mass of gastric tumors in *LysM*^+/+^;*Myc*^fl/fl^;*Gp130*^F/F^ and *LysM*^Cre/+^;*Myc*^fl/fl^;*Gp130*^F/F^ mice collected at 100 days of age. Each symbol represents an individual mouse. n = 12 mice per group. (**C**) *Myc* gene expression in subcutaneous KPT gastric tumor organoids of *LysM*^+/+^;*Myc*^fl/fl^ and *LysM*^Cre/+^;*Myc*^fl/fl^ hosts. n = 4 mice per group. (**D**) Mass of subcutaneous KPT gastric tumor organoids from *LysM*^+/+^;*Myc*^fl/fl^ and *LysM*^Cre/+^;*Myc*^fl/fl^ hosts. Mice were collected at 3 weeks following tumor organoid injection. Each symbol represents an individual mouse. n ≥ 4 mice per group. (**E**) *Myc* gene expression in TAMs isolated from subcutaneous B16F10 tumors of *LysM*^+/+^;*Myc*^fl/fl^ and *LysM*^Cre/+^;*Myc*^fl/fl^ hosts. n ≥ 3 mice per group. (**F**) Mass of subcutaneous B16F10 tumors from *LysM*^+/+^;*Myc*^fl/fl^ and *LysM*^Cre/+^;*Myc*^fl/fl^ hosts. Mice were collected at 2 weeks following tumor cell injection. Each symbol represents an individual mouse. n = 11 mice per group. (**G**) *Myc* gene expression in TAMs isolated from subcutaneous MC38 tumors of *LysM*^+/+^;*Myc*^fl/fl^ and *LysM*^Cre/+^;*Myc*^fl/fl^ hosts. n ≥ 3 mice per group. (**H**) Mass of subcutaneous MC38 tumors from *LysM*^+/+^;*Myc*^fl/fl^ and *LysM*^Cre/+^;*Myc*^fl/fl^ hosts. Mice were collected at 2 weeks following tumor cell injection. Each symbol represents an individual mouse. n ≥ 13 mice per group. Data represent mean ± SEM; * *p* < 0.05, ** *p* < 0.01, *** *p* < 0.001, with statistical significance determined by an unpaired Student’s *t*-test.

To assess the contribution of Myc signaling in macrophages to the growth of tumors with low immunogenicity, we crossed *LysM*^+/+^;*Myc*^fl/fl^ and *LysM*^Cre/+^;*Myc*^fl/fl^ mice to the *Gp130*^F/F^ mouse model. In *Gp130*^F/F^ mice, a disruption of the Socs3-dependent negative feedback loop on the shared IL-6 cytokine family gp130 receptor subunit results in excessive STAT3 signaling that promotes the spontaneous development of gastric adenomas from 6 weeks of age [28,29]. Owing to the ontogenetic relationship between TAMs and bone-marrow-derived macrophages, we first confirmed reduced Myc expression in tumors and TAMs of *LysM*^+/+^;*Myc*^fl/fl^;*Gp130*^F/F^ mice (Figure 2A and Supplementary Figure S1A,B). However, we did not observe a difference in tumor burden between *LysM*^+/+^;*Myc*^fl/fl^;*Gp130*^F/F^ and *LysM*^Cre/+^;*Myc*^fl/fl^;*Gp130*^F/F^ littermates (Figure 2B). By contrast, tamoxifen-induced Cre-mediated ablation of *Myc* in the gastric epithelium of tumor-bearing *Tff1*^CreERT2^;*Myc*^fl/fl^;*Gp130*^F/F^ compound mutant mice significantly reduced gastric tumor burden (Supplementary Figure S1C,D).

Because our observations suggested that *Myc* expression in TAMs may not affect tumor growth during early adenomatous stages, we next determined whether *Myc* ablation in TAMs affected the growth of tumors that develop into invasive carcinomas. For this, we established subcutaneous tumors in *LysM*^+/+^;*Myc*^fl/fl^ and *LysM*^Cre/+^;*Myc*^fl/fl^ hosts using tumor organoids derived from invasive gastric adenocarcinomas of *Kras*^G12D/+^;*Pik3ca*^H1047R/+^;*Trp53*^R172H/+^ (KPT) mice. We confirmed reduced *Myc* expression in KPT tumors excised from *LysM*^Cre/+^;*Myc*^fl/fl^ hosts; however, these tumors were of a comparable size to those collected from Myc-proficient *LysM*^+/+^;*Myc*^fl/fl^ hosts (Figure 2C,D).

To ascertain whether our observations remained pertinent to gastric tumors, we next assessed the contribution of myeloid cell-specific *Myc* depletion in hosts engrafted with highly immunogenic B16F10 melanoma or MC38 colon cancer allografts. Despite an 80% reduction in *Myc* expression in *LysM*^Cre/+^;*Myc*^fl/fl^ TAMs, we could not detect differences in the growth of B16F10 tumors between *LysM*^+/+^;*Myc*^fl/fl^ and *LysM*^Cre/+^;*Myc*^fl/fl^ hosts (Figure 2E,F). In contrast, reduced *Myc* expression in MC38 TAMs coincided with larger tumors in *LysM*^Cre/+^;*Myc*^fl/fl^ hosts compared to their *LysM*^+/+^;*Myc*^fl/fl^ littermates (Figure 2G,H). Collectively, our results suggest that *Myc* ablation in host macrophages does not attenuate tumor growth regardless of immunogenicity, the cellular origin of the tumor, the site it develops, or the stage along the tumor progression cascade.

### 3.3. Conditional Ablation of Myc in TAMs Neither Reduces Tumor Immune Suppression Nor Impairs Alternative Macrophage Polarization

**Figure 3:**
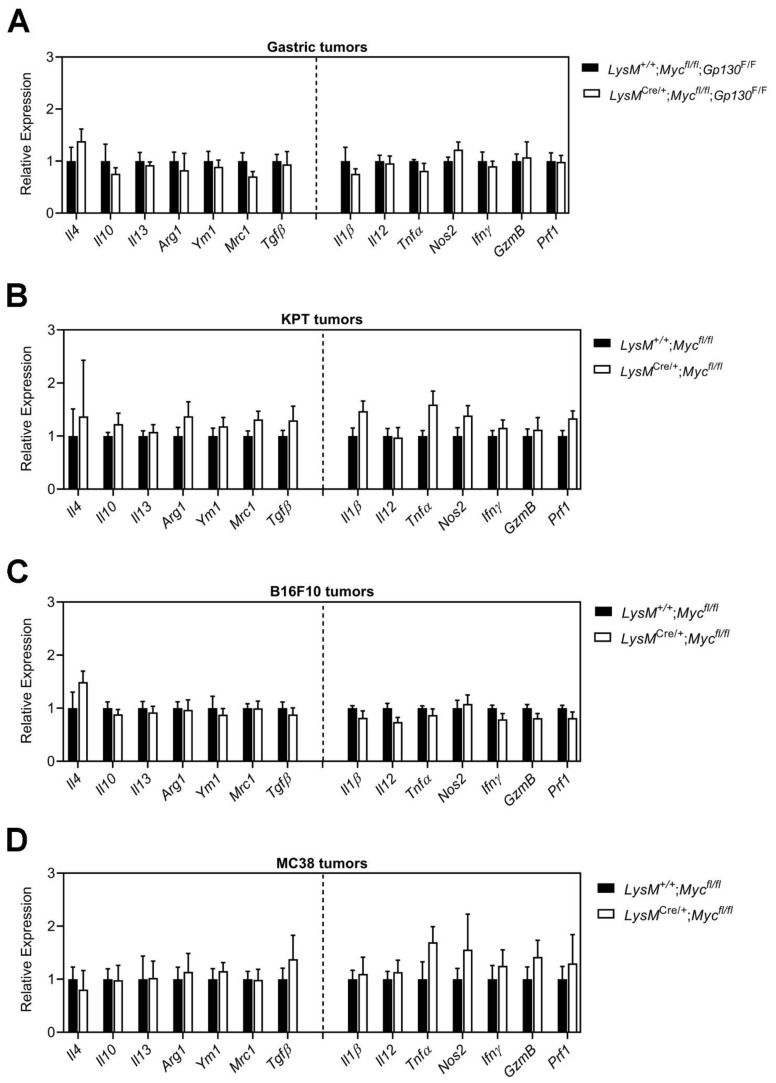
**Inhibition of Myc signaling in TAMs does not reduce tumor immune suppression.** qPCR analysis on (**A**) *Gp130*^F/F^, (**B**) KPT, (**C**) B16F10 and (**D**) MC38 tumors of *LysM*^+/+^;*Myc*^fl/fl^ and *LysM*^Cre/+^;*Myc*^fl/fl^ hosts for genes associated with immune suppression (i.e., *Il4, Il10, Il13, Arg1, Ym1, Mrc1, Tgfβ*) or immune activation (i.e., *Il1β, Il12, Tnfα, Nos2, Ifnγ, GzmB, Prf1*). n = 4 mice per group. Data represent mean ± SEM.

*Myc* expression in TAMs has been shown to regulate tumor growth by reinforcing an immunosuppressive microenvironment [13,14,30], while the inhibition of Myc enables T-cell-mediated immune surveillance [31,32]. We therefore profiled tumors from *Gp130*^F/F^, KPT, B16F10, or MC38 models that arose in either *LysM*^+/+^;*Myc*^fl/fl^ or *LysM*^Cre/+^;*Myc*^fl/fl^ hosts for markers associated with immune suppression (i.e., *Il4*, *Il10*, *Il13*, *Arg1*, *Ym1*, *Mrc1*, *Tgfβ*) and immune activation (i.e., *Il1β*, *Il12*, *Tnfα*, *Nos2*, *Ifnγ*, *GzmB* and *Prf1*). Assessment of the corresponding transcripts by qPCR across all four models showed comparable gene expression levels in tumors irrespective of the genotype of the hosts (Figure 3A–D).

**Figure 4:**
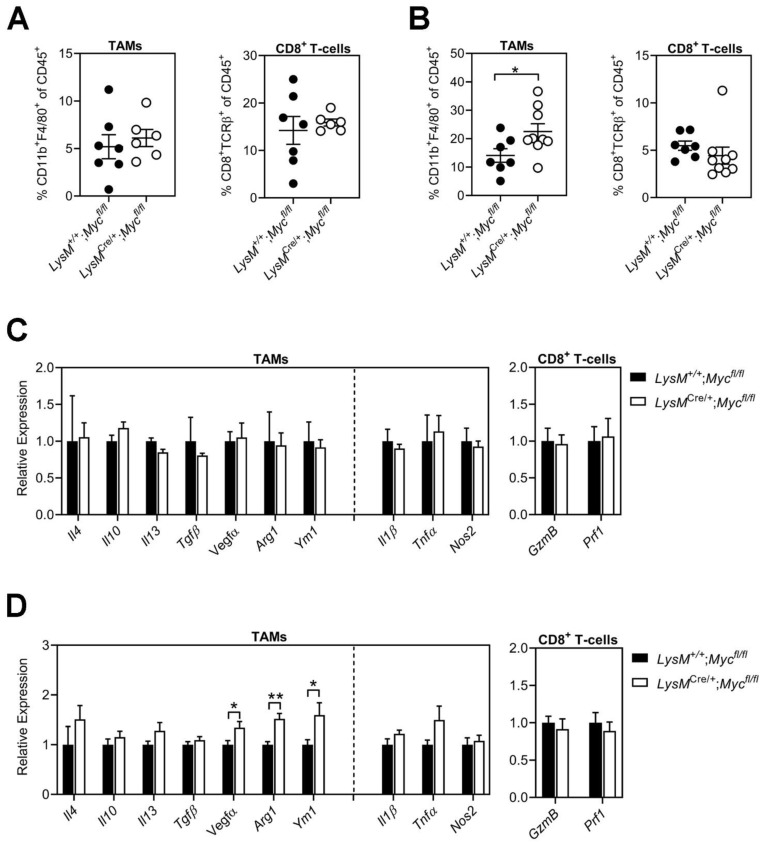
**Genetic reduction of *Myc* in TAMs does not inhibit alternative macrophage polarization or stimulate adaptive anti-tumor immunity.** (**A**,**B**) Quantification of TAMs and CD8^+^ T-cells in subcutaneous (**A**) B16F10 and (**B**) MC38 tumors of *LysM*^+/+^;*Myc*^fl/fl^ and *LysM*^Cre/+^;*Myc*^fl/fl^ hosts. Each symbol represents an individual mouse. n ≥ 6 mice per group. (**C**,**D**) qPCR analysis on CD45^+^CD11b^+^F4/80^+^Ly6C^−^LygG^−^ TAMs and CD45^+^TCRβ^+^CD8^+^ T-cells isolated from subcutaneous (**C**) B16F10 and (**D**) MC38 tumors of *LysM*^+/+^;*Myc*^fl/fl^ and *LysM*^Cre/+^;*Myc*^fl/fl^ hosts. n ≥ 3 mice per group. Data represent mean ± SEM; * *p* < 0.05, ** *p* < 0.01, with statistical significance determined by an unpaired Student’s *t*-test.

To investigate whether in vivo depletion of *Myc* in macrophages resulted in more subtle changes in TAM and CD8^+^ T-cell recruitment and activation, we performed flow cytometry to quantify the proportion of these immune cells in B16F10 and MC38 tumors established in *LysM*^+/+^;*Myc*^fl/fl^ and *LysM*^Cre/+^;*Myc*^fl/fl^ hosts. We observed a comparable abundance of TAMs and CD8^+^ T-cells in B16F10 tumors between *LysM*^+/+^;*Myc*^fl/fl^ and *LysM*^Cre/+^;*Myc*^fl/fl^ hosts (Figure 4A). In line with the increased tumor burden observed with MC38 tumor cells in *LysM*^Cre/+^;*Myc*^fl/fl^ hosts, we also observed an increased proportion of TAMs in these tumors compared to *LysM*^+/+^;*Myc*^fl/fl^ controls. However, this difference did not impact the abundance of tumor-infiltrating CD8^+^ T-cells, which remained similar across both groups (Figure 4B).

We next purified TAMs from subcutaneous B16F10 and MC38 tumors established in *LysM*^Cre/+^;*Myc*^fl/fl^ hosts to determine whether their endotype would differ from TAMs associated with tumors from *LysM*^+/+^;*Myc*^fl/fl^ hosts. We assessed gene signatures comprising prototypical markers for classical (i.e., *Il1β*, *Tnfα*, *Nos2*) and alternative macrophage activation (i.e., *Il4*, *Il10*, *Il13*, *Tgfβ, Vegfα, Arg1*, *Ym1*). We did not detect significant differences for any of these markers between TAMs isolated from B16F10 tumors of *LysM*^Cre/+^;*Myc*^fl/fl^ and *LysM*^+/+^;*Myc*^fl/fl^ hosts (Figure 4C). By contrast, we observed increased expression of genes associated with alternative macrophage polarization (e.g., *Vegfα, Arg1*, *Ym1*) in TAMs isolated from MC38 tumors of *LysM*^Cre/+^;*Myc*^fl/fl^ hosts, while the expression of genes associated with classical macrophage polarization remained unchanged (Figure 4D).

To assess whether the increased MC38 tumor burden and alternatively activated endotype of TAMs observed in *LysM*^Cre/+^;*Myc*^fl/fl^ hosts could be attributed to reduced activity of CD8^+^ effector T-cells, we next assessed the expression of cytotoxic molecules (i.e., *GzmB* and *Prf1*) in CD8^+^ T-cells isolated from MC38 tumors of *LysM*^Cre/+^;*Myc*^fl/fl^ and *LysM*^+/+^;*Myc*^fl/fl^ hosts (Figure 4D). Surprisingly, we did not observe a difference in *GzmB* or *Prf1* expression between CD8^+^ T-cells isolated from MC38 tumors between groups, suggesting that impaired T-cell mediated anti-tumor immune responses are unlikely to explain the increased tumor burden observed in *LysM*^Cre/+^;*Myc*^fl/fl^ hosts.

## 4. Discussion

In this study, we provide comprehensive evidence across complementary early adenomatous and carcinoma models that genetic ablation of *Myc* in TAMs fails to confer increased host-mediated anti-tumor responses. Because the contribution of TAMs to tumorigenesis can be mediated by affecting the recruitment and/or activation of effector T-cells, we also confirmed that *Myc*-depleted macrophages did not improve host anti-tumor immune responses in highly immunogenic tumors. We surmise from our data that systemic targeting of Myc is unlikely to confer tumor cell-extrinsic therapeutic benefits that arise from direct modulation of TAM polarization.

Macrophages constantly alter their endotype in response to their surroundings, which include signals derived from pathogens and immune cells [2,33,34]. This allows them to orchestrate diverse activities, including stimulating host immunity, removal of cellular debris, and wound healing. Classically activated M1 macrophages are activated following exposure to bacterial lipopolysaccharide or Th1-associated cytokines (e.g., TNFα and IFNγ), and exhibit a pro-inflammatory and phagocytic endotype [35,36]. In contrast, alternatively activated M2 macrophages play a pivotal role in immune suppression, wound healing, and fibrosis, and are induced by parasitic infections, immune-suppressive cytokines (e.g., IL4, IL10, IL13), and glucocorticoids [35,36]. Given the broad range of environmental cues dictating macrophage endotypes, transcription factors including peroxisome proliferation-factor receptors (PPARs) [37], signal transducer and activator of transcription (STAT)s [38,39], CAATT enhancer-binding proteins [40], interferon regulatory factors (IRFs) [41], Kruppel-like factors (KLF) [42], and NF-κB [43] have been shown to functionally polarize macrophages. Moreover, alterations to signaling molecules upstream of these transcription factors, including CSF1R [44], PI3Kγ [45], or the SRC kinase HCK [23,25,26,46], are currently exploited to limit alternative macrophage polarization as adjuvant therapy for cancer.

Myc has been previously implicated as part of the signaling cascade that affects macrophage polarization [47]; however, these observations remain conflicting. For example, the inhibition of Myc suppresses HIF1α, a key metabolic regulator of classical M1 macrophage polarization [48,49,50]. In contrast, degradation of Myc by the E3 ligase FBXW7 restricts alternative M2 TAM polarization and limits tumor progression [51]. In another study, Myc activity enhanced M2 macrophage polarization by transcriptionally repressing miR-26a [52,53]. Finally, Pello and colleagues observed that genetic ablation of *Myc* using *LysM*^Cre/+^;*Myc*^fl/fl^ hosts reduced the growth of subcutaneous B16F10 tumors and correlated these in vivo findings with a bias away from an alternatively activated gene expression signature in TAMs [13,14].

Surprisingly, our observations have failed to substantiate the observation of impaired B16F10 tumors in *LysM*^Cre/+^;*Myc*^fl/fl^ hosts. Importantly, we also failed to document increased anti-tumor activity despite less than 20% residual *Myc* expression in TAMs or tumors of *LysM*^Cre/+^;*Myc*^fl/fl^ hosts in the MC38 and KPT allograft models, as well as in the endogenous *Gp130*^F/F^ adenoma model. Although phenotypic differences have been described across the various conditional *Myc* alleles [17,54], we note that in their studies Pello and colleagues [13,14] used the same *LysM*^Cre/+^ transgene (*Lyz2*^tm1(cre)Ifo^) [16] and *Myc*^fl/fl^ allele (*Myc*^tm2Fwa^) [17] as we used for our assessment. These contrasting findings may be attributed to differences in the genetic background of the mouse models, the microbiome, and other less controllable (e.g., environmental) influences between the different studies. However, the identification of these parameters remains challenging because the exact molecular mechanism by which myeloid-specific *Myc* ablation affects tumor growth remains unknown over and above a “guilt by association” correlation with the extent of M2 macrophage polarization. Indeed, we were unable to correlate the differences observed between MC38 tumor allografts in *LysM*^+/+^;*Myc*^fl/fl^ and *LysM*^Cre/+^;*Myc*^fl/fl^ hosts with either altered abundance or activity of cytotoxic T-cells, suggesting that the increased tumor burden in *LysM*^Cre/+^;*Myc*^fl/fl^ hosts is unlikely to result from reduced adaptive anti-tumor immunity. These observations are in striking contrast to the observation that excessive Myc activity in Kras-driven lung adenomas accelerates adenocarcinoma development as a result of an immune-suppressed stroma. The latter arises from instructive signals by tumor-derived factors (i.e., CCL9 and IL23) that attract TAMs and mediate the exclusion of effector immune cells, respectively [55].

In summary, our collective insights from our models of substantial Cre/lox-mediated ablation of *Myc* in macrophages suggest that Myc is unlikely to serve as a reliable therapeutic anti-tumor target in these host cells. This is in stark contrast to the reduced tumor burden observed in models of Cre/lox-mediated *Myc* ablation in tumor cells, including our own observations in *Tff1*^CreERT2^;*Myc*^fl/fl^;*Gp130*^F/F^ *Myc-*depleted gastric adenomas. Thus, we predict that the therapeutic administration of systemic-acting anti-Myc therapies in humans may confer their main therapeutic effect directly on cancer cells, rather than by directly reinvigorating anti-tumor immunity. Indeed, our observations suggest careful assessment, as some tumors may thrive upon the inhibition of Myc in myeloid cells. In-depth comparison between treatment response and cellular analysis in ongoing phase I/II trials with the Myc-antagonist Omomyc/OMO-103 in patients with advanced solid malignancies (NCT04808362) will ultimately identify the mechanisms that underpin the clinical impact of Myc inhibition.

## Data Availability

The data presented in this study are available on request from the corresponding author Matthias Ernst (matthias.ernst@onjcri.org.au).

## Supplementary Materials

The following supporting information can be downloaded at: https://www.mdpi.com/article/10.3390/cells11244104/s1, Figure S1: Epithelial-specific ablation of *Myc* reduces gastric tumor growth in *Gp130*^F/F^ mice.
