# Supplementary material for: Tumor Growth Remains Refractory to Myc Ablation in Host Macrophages
Source: Cells. 2022 Dec 17;11(24):4104. doi: 10.3390/cells11244104 (PMC9777527; doi:10.3390/cells11244104)
Supplement: Supplementary file 1 [file cells-11-04104-s001.zip › cells-2071430-supplementary.pdf]

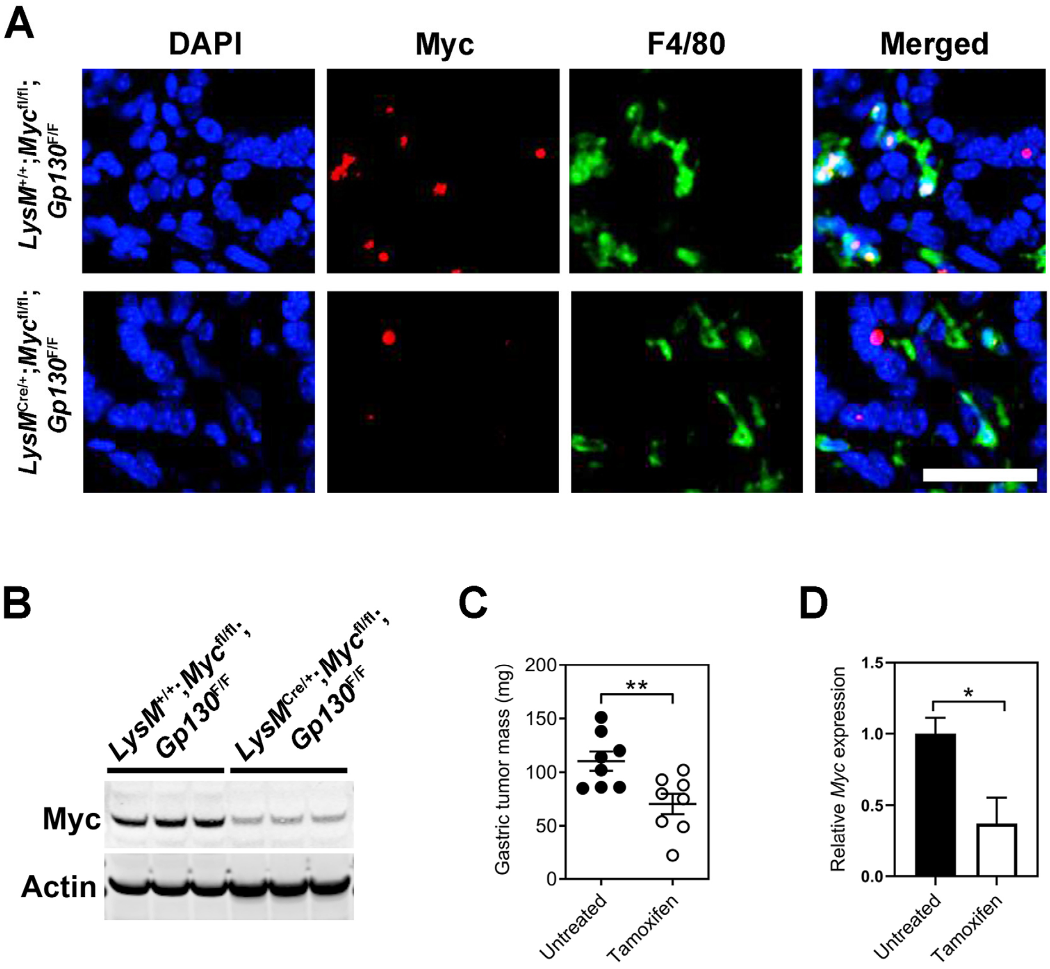

Supplementary Figure S1. Epithelial-specific ablation of *Myc* reduces gastric tumor growth in $Gp130^{F/F}$ mice. (**A**) Representative immunofluorescence staining for Myc and F4/80^{+} TAMs in gastric tumors of $LysM^{+/+};Myc^{fl/fl};Gp130^{F/F}$ and $LysM^{Cre/+};Myc^{fl/fl};Gp130^{F/F}$ mice. DAPI was used to visualize nuclei. Scale bar: 50 µm. (**B**) Western blot analysis of Myc expression in gastric tumors of $LysM^{+/+};Myc^{fl/fl};Gp130^{F/F}$ and $LysM^{Cre/+};Myc^{fl/fl};Gp130^{F/F}$ mice. Actin was used as a loading control. Each lane represents an individual mouse. (**C**) Mass of gastric tumors in $Tff1^{CreERT2};Myc^{fl/fl};Gp130^{F/F}$ mice following treatment with tamoxifen to ablate Myc expression in stomach epithelial cells. Each symbol represents an individual mouse. n = 8 mice per group. (**D**) *Myc* gene expression in gastric tumors of $Tff1^{CreERT2};Myc^{fl/fl};Gp130^{F/F}$ mice following treatment with tamoxifen (1mg/mL per dose; 2 doses a day over 3 consecutive days) to ablate *Myc* expression in stomach epithelial cells. Mice were euthanized 7 days following the last tamoxifen injection. $n \geq 6$ mice per group. Data represent mean ± SEM; * $p < 0.05$, ** $p < 0.01$, with statistical significance determined by an unpaired Student's *t*-test.
